# Genetic (KIR, HLA-C) and Some Clinical Parameters Influencing the Level of Liver Enzymes and Early Virologic Response in Patients with Chronic Hepatitis C

**DOI:** 10.1007/s00005-015-0350-1

**Published:** 2015-07-24

**Authors:** Iwona Mozer-Lisewska, Katarzyna Zwolińska, Arleta Elżbieta Kowala-Piaskowska, Maciej Bura, Błażej Rozpłochowski, Anna Pauli, Jan Żeromski, Egbert Piasecki, Piotr Kuśnierczyk

**Affiliations:** Chair and Department of Infectious Diseases, Karol Marcinkowski University of Medical Sciences, Poznan, Poland; Laboratory of Virology, Department of Immunology of Infectious Diseases, Ludwik Hirszfeld Institute of Immunology and Experimental Therapy, Polish Academy of Sciences, ul. Rudolfa Weigla 12, 53-114 Wrocław, Poland; Chair of Clinical Immunology, Karol Marcinkowski University of Medical Sciences, Poznan, Poland; Laboratory of Immunogenetics and Tissue Immunology, Ludwik Hirszfeld Institute of Immunology and Experimental Therapy, Polish Academy of Sciences, ul. Rudolfa Weigla 12, 53-114 Wrocław, Poland

**Keywords:** *KIR2DS4*, HLA-C C2, ALT, AST, HCV chronic infection, Early virologic response

## Abstract

Natural killer cells play an important role as effectors of innate immunity and regulators of adaptive immunity. They are important elements of the innate response to viral infections, which they detect using human leukocyte antigen (HLA) class I-binding receptors. Most polymorphic of these are killer cell immunoglobulin-like receptors (KIRs) which exist as two basic isotypes, activating or inhibitory receptors and are encoded by genes distributed differently in unrelated individuals. We searched for links between selected clinical data (including HCV viremia, liver enzymes level and liver histology parameters) and the presence of genes encoding these receptors and their ligands in hepatitis C virus-infected individuals subjected to pegylated interferon-α and ribavirin therapy. Genomic DNA samples from two hundred and ninety-two chronically infected patients were typed by polymerase chain reaction for the presence or absence of genes for KIRs and their ligands, class I HLA molecules, and clinical data of the patients were collected. Our results suggest an importance of clinical parameters and the contribution of KIR and HLA genes to the course of hepatitis C virus infection and the response to therapy. The study revealed that levels of liver enzymes before therapy were about 30 % higher in patients who possessed a variant *KIR2DS4* gene with 22-base pair deletion. Decrease of ALT activity after treatment was higher in *HLA*-*C C2*-positive than negative individuals. Beside it, patients demonstrated early virologic response to the therapy if the time lag before treatment was short, particularly in women.

## Introduction

Hepatitis C virus (HCV) has a single-stranded RNA genome and belongs to *Flaviviridae* family. The viral genome is highly heterogenous encompassing six genotypes and their various subtypes. Its rapid replication is characterized by tremendous variability due to having an error-prone RNA polymerase, such that during synthesis of new RNA, there is no proofreading of the newly synthesized strand and multiple quasispecies can then be readily generated (Freeman et al. 2001). It is one of the reasons why an HCV vaccine is still not available. About 750,000 people are chronically infected with HCV in Poland (Bura et al. 2012). The outcome of chronic infection depends on HCV genotype, viral kinetics, immune response in patient, anti-viral treatment, as well as other factors (Irshad et al. 2008; Layden-Almer and Layden 2003). A favorable outcome in HCV infection is considered to be a sustained virologic response (SVR), manifested as a lack of detectable HCV RNA in the patient serum directly after completion of the antiviral therapy and 24 weeks later, verified by the qualitative test. This result is achieved, however, in about 50 % of patients only (Asselah et al. 2010; Ghany et al. 2009). Relatively poor effectiveness of the pharmacological treatment directed the attention and effort of many researchers toward understanding the role of the immune system in determining the outcome of HCV infection. We are aware of the fact that modern drugs nowadays such as direct antiviral agents have revolutionized the management of hepatitis C. Nevertheless, classical interferon (IFN)-ribavirin combination therapy is still used worldwide due to its accessibility, cost-effectiveness and other factors.

It has been shown that an useful tool for predicting the outcome of therapy is also early virologic response (EVR), defined as at least 100-fold (2 log_10_) decrease of concentration of HCV-RNA after 12 weeks of the treatment compared to pre-therapeutic value (Bura et al. 2012). Viral infections are controlled by innate immunity at the first instance, and by adaptive immunity which develops later (Brenndörfer and Sällberg 2012; Żeromski et al. 2011). Efficient innate response to viruses is exerted by natural killer (NK) cells. These cells can detect virally infected hepatocytes due to their receptors, the most polymorphic of which are killer immunoglobulin-like receptors (KIRs). These receptors, after binding appropriate ligands, either activate or inhibit NK cells. Virally infected cells frequently express much lower amounts of human leukocyte antigen (HLA) class I molecules than non-infected ones, which makes them vulnerable to the attack of NK cells if their KIRs do not detect some HLA class I molecules on their targets (Kuśnierczyk 2013; Żeromski et al. 2011). The aim of this study was to search for links between several clinical, virologic and biochemical data and the presence of *KIR* genes and their ligands in a large cohort of HCV-infected individuals subjected to antiviral therapy.

## Materials and Methods

### Study Subjects

We studied 292 (135 women and 157 men) HCV-infected individuals whose clinical characteristics are given in Table 1. All patients were Polish Caucasians from western Poland and had a chronic HCV infection, diagnosed according to generally accepted criteria. Serum HCV-RNA viral load was assessed by RT-PCR Amplicor HCV™ version 2.0 (Roche Diagnostics, Germany); sensitivity level, 50 IU/ml (qualitative assessment) and 600 IU/ml (quantitative assessment). The HCV genotype was established using VERSANT HCV Genotype 2,0 Assay, LiPA (Siemens Healthcare Diagnostics, Poznań, Poland).

**Table 1:** Clinical characteristics of patients

Feature	Median	*S*_*n*_	*Q*_1_	*Q*_3_	95 % CI	
Age at infection	38	13	24	50	36	39
Viremia (×10^3^) before treatment	42.8	41.76	11.6	127	28.9	54.8
ALT before treatment	47.9	26	33.5	80.38	43.6	54
ALT after treatment	28.6	15.9	18	49.6	23.1	32
AST before treatment	39.2	17.2	25	56.22	36.5	42.3
Bilirubin before treatment	0.77	0.25	0.52	0.98	0.72	0.83
AFP before treatment	4.64	2.56	2.87	7.77	4.1	5
GGTP before treatment	40.9	27.1	20.45	70	33.9	45.6

		Liver histology			
Feature		*n*	%	95 % CI	
Inflammation	0	3	1.94	0.4	5.55
	1	45	29.03	22.03	36.86
	2	82	52.9	44.73	60.96
	3	24	15.48	10.18	22.16
	4	1	0.65	0.02	3.54
Fibrosis	0	14	7.07	3.92	11.58
	1	87	43.94	36.91	51.15
	2	51	25.76	19.82	32.44
	3	26	13.13	8.76	18.65
	4	20	10.1	6.28	15.17
Cirrhosis	Yes	30	10.27	7.04	14.34
	No	262	89.73		

		Comorbidities			
Feature		*n*	%	95 % CI	
Metabolic syndrome	Yes	76	26.03	21.09	31.46
	No	216	73.97		
Thyroid diseases	Yes	16	5.48	3.16	8.75
	No	276	94.52		
CNS diseases	Yes	9	3.08	1.42	5.77
	No	283	96.92		
Diseases of cardiovascular system	Yes	15	5.14	2.9	8.33
	No	277	94.86		
Diseases of urogenital system	Yes	15	5.14	2.9	8.33
	No	277	94.86		
Neoplasm	Yes	24	8.22	5.34	11.98
	No	268	91.78		

*AFP* alphafetoprotein, *S*_*n*_ average dispersion, *GGTP* gammaglutamylotranspeptidase, *Q*_*1*_, *Q*_*3*_ first and third quartiles, respectively

Liver enzymes alanine transferase (ALT) and aspartate transferase (AST) activities, as well as gammaglutamylotranspeptidase were assessed by an enzymatic method, using the MURA 200 (Pointe Scientific, Italy) analyser with their reagents and calibrators (upper normal limit 40 IU/L). Alphafetoprotein content was performed by means of chemiluminescence assay on ARCHITECT 2000 analyser (Abbott, USA).

All patients were subjected to percutaneous liver needle biopsy. Paraffin tissue sections were assessed by experienced pathologist and the assessment included the evaluation of inflammatory activity—grading and spread of fibrosis in the range of 0–4 scores.

Patients were treated with pegylated IFN-α and ribavirin in doses adjusted to their body mass, as described earlier (Bura et al. 2012). EVR was defined by at least 100-fold decrease of HCV-RNA concentration in serum after 12 weeks of the treatment compared to pretreatment value.

Not all clinical parameters were available for all patients, which is reflected in numbers given in tables.

Informed consent was obtained from each patient included in the study. The study protocol conforms to the ethical guidelines of the 1975 Declaration of Helsinki as reflected in a priori approval by the Karol Marcinkowski University of Medical Sciences Bioethical Committee.

### DNA Isolation and KIR and HLA Typing

Genomic DNA of examined individuals was isolated from peripheral blood using QIAamp^®^ DNA Blood Mini Kit (Qiagen, Hilden, Germany), and *KIR* genes and *HLA*-*C C1* and *C2* as well as *HLA*-*B* and *HLA*-*A Bw4* and *Bw6* allotypes were typed by means of sequence-specific primers in PCR. Briefly, *KIR* genotyping was performed using primers according to Vilches et al. (2007) with our modifications (Kuśnierczyk et al. 2015). *HLA*-*A*, *HLA*-*B* and *HLA*-*C* allotypes were detected using commercially available tests (Olerup SSP^®^ KIR HLA Ligand, Olerup GmbH, Sweden) using Taq DNA Polymerase (Qiagen, Germany).

### Statistical Analysis

Global test for the difference between two sets of *k* dependent proportions i.e. $x_{1} = \left( {p_{11} ,\; \ldots ,p_{1k} } \right)^{T} {\text{and}}\;{\mkern 1mu} x_{2} = \left( {p_{21} , \ldots ,p_{2k} } \right)^{T}$ was $T = \frac{{\left\| {x_{1} - \left. {x_{2} } \right\|_{2} } \right.}}{{{\text{SE}}_{{\left\| {x_{1} - x_{2} } \right\|_{2} }} }}$, where distribution of *T* statistic was estimated with Monte Carlo method. Akaike’s information criterion was used as a measure of fit of generalized linear models. Chi square test for two proportions and exact test, if necessary, were used for cross-classification tables. Odds ratio (OR) and its 95 % confidence interval were used as a measure of effect size. The confidence intervals for the differences of two independent proportions were estimated with Agresti-Caffo method. Viremia was expressed as logs of viral RNA copy number/ml/10^3^, AST and ALT were expressed as logs of IU/L and linear models were performed to investigate relations between viremia, AST, ALT and genetic factors adjusted for clinical and anthropological characteristics. The *S*_*n*_ statistic was computed as the measure of variability: $S_{n} = {\text{med}}\left\{ {{\text{med}}\left| {x_{i} - x_{j} } \right|;{\mkern 1mu} j = 1 \ldots n} \right\}$ (Rousseeuw and Croux 1993). Statistics and their confidence intervals were estimated with bootstrap approach if necessary.

## Results

Our patients were HCV-infected most frequently in middle age (Table 1; median: 38 years). Their median viremia value was 42.8 × 10^3^ copies/ml, whereas 25 % of them (Q1) had not more than 11.6 × 10^3^ copies/ml, and another 25 % (Q3) had above 127 × 10^3^ copies/ml (Table 1). Majority of patients were infected with HCV genotype 1b, and minor fractions with other genotypes (Table 2). Nine of the 14 mixed infections included only genotypes 4a, 4b, 4c, 4d and/or 4e.

**Table 2:** HCV genotype frequencies in patients. ^a^ HCV genotypes in 57 patients were not determined

Genotype	*n*	%	95 % CI	
1a	17	6.97	4.11	10.92
1b	191	78.28	72.57	83.29
3	18	7.38	4.43	11.41
4	4	1.64	0.45	4.14
Mixed	14	5.74	3.17	9.44
Total	244^a^	100	–	–

**Fig. 1:**
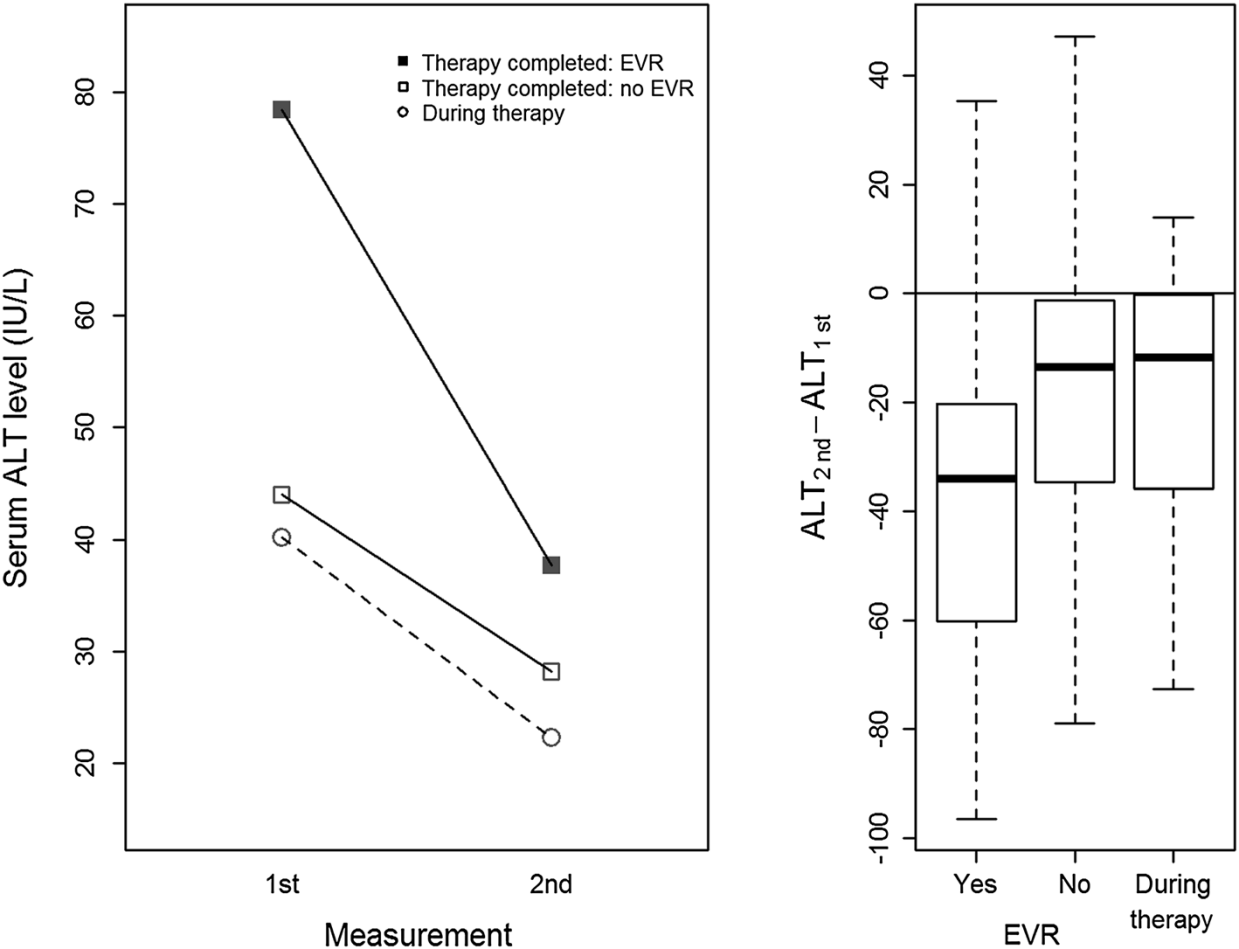
ALT levels before and after therapy in patients presenting early virologic response (EVR) or without such a response

**Fig. 2:**
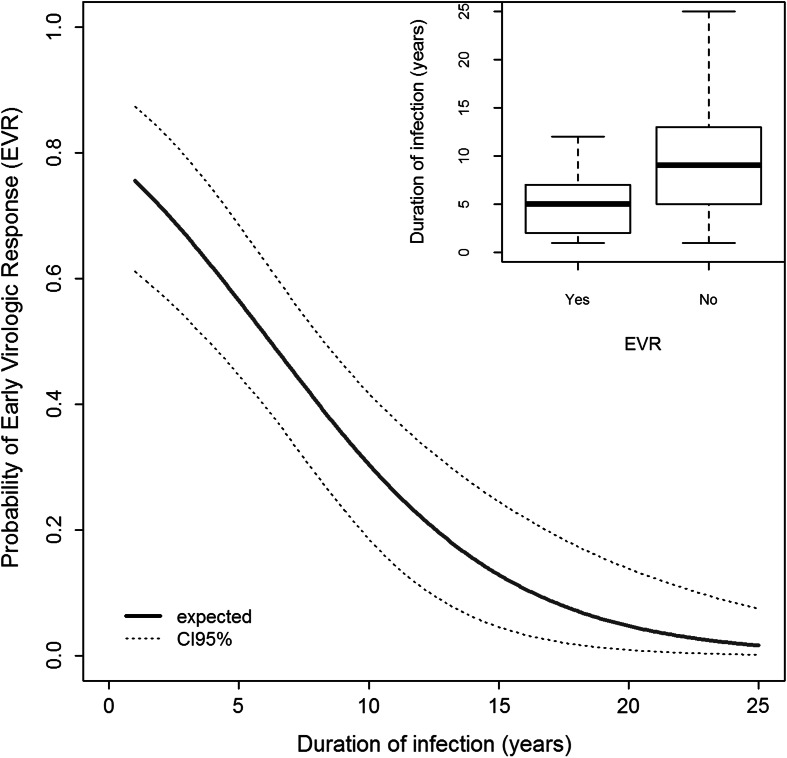
Probability of early virologic response (EVR) depending on duration of untreated HCV infection. Insert: medians, first and third quartiles duration of untreated infection in patients positive and negative for EVR

**Fig. 3:**
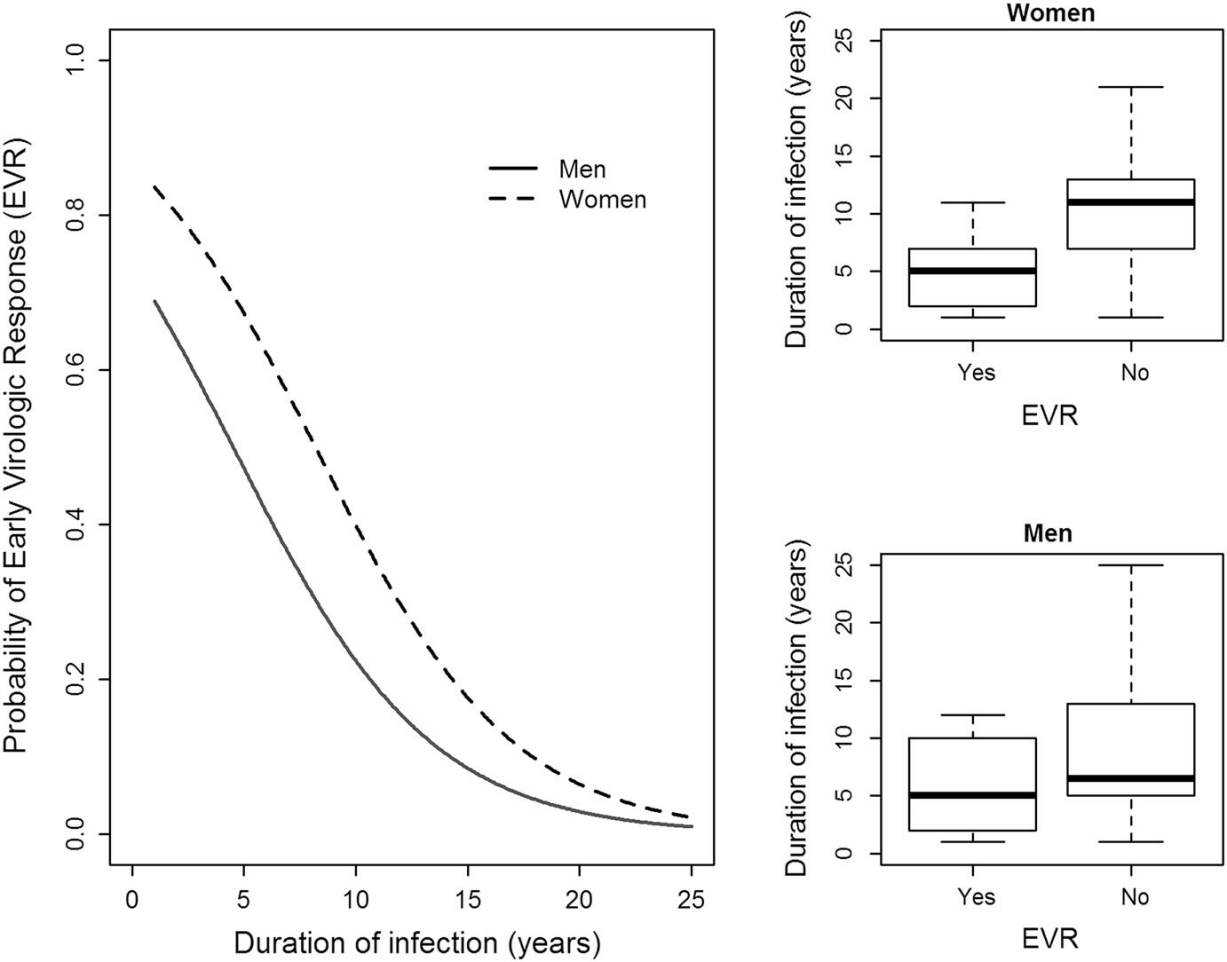
Comparison of the probability of early virologic response (EVR) depending on duration of untreated HCV infection in men and women. *Left panel* comparison of EVR probability in men and women depending on the duration of infection. *Right upper panel* medians, 1st and 3rd quartiles duration of infection of EVR-positive and EVR-negative women. *Right lower panel* medians, first and third quartiles duration of infection of EVR-positive and EVR-negative men

Median ALT values before therapy with pegylated IFN-α and ribavirin were about 47.9 IU/L, but dropped down to 28.6 after the therapy. Roughly half of the patients displayed an EVR after therapy, whereas the other half required re-therapy (Table 3). ALT levels before therapy were higher in patients who later exhibited EVR than in non-responders, but were reduced twice after therapy (Fig. 1). In contrast, patients who did not reached an EVR, had lower ALT levels before therapy, but these were reduced for only 36 % after therapy (Fig. 1). The EVR was dependent on the duration of infection before therapy: a median time of infection was 5 years in EVR-positive patients and 9 years in EVR-negative ones (Fig. 2, insert). The probability of EVR decreased dramatically with elongation of time of untreated infection (20 % each year) with virtually no chance of response in patients infected 25 years before treatment (Fig. 2). This probability was higher in women with short time of untreated infection than in men with the same infection time, but leveled to the same low value in both sexes when infected for 25 years (Fig. 3). Patient’s age, sex, and the presence of cirrhosis were also strongly associated with AST and ALT levels before therapy (Table 4).

**Table 3:** Factors influencing the results of therapy measured by early virologic response (EVR = 1) or its lack (EVR = 0, necessity of re-therapy). ^a^ EVR was evaluated for 102 of 292 patients. Twenty-six individuals with EVR achieved also sustained virologic response (SVR), other patients are waiting 6 months after completion of treatment to be searched for SVR

Patients under therapy (*n* = 102)^a^	Success of therapy (EVR = 1)	Re-treatment (EVR = 0)
*n*	47	55
%	46.08	53.92
95 % CI	36.16; 56.23	43.77; 64.84
Duration of uncured infection (years)		
Median	5	9
*S*_*n*_	3	5
95 % CI	4; 6	6; 12
OR 0.802	95 % CI 0.719; 0.877	*p* = 0.000033
Gender (female)		
OR 2.395	95 % CI 0.94; 6.73	*p* = 0.072

*OR* odds ratio

**Table 4:** Factors associated with AST and ALT levels before therapy. ^a^ Changes in AST and ALT level per each 3 years of life

First measurement	Factors	Beta (%)	95 % CI		*p*
AST (Log IU/L)	Patient’s age	1.4^a^	0.92	1.87	0.00001
	Gender (female)	−14.62	−25.81	−2.14	0.02541
	Cirrhosis	68.69	27.97	118.67	0.00018
	*KIR2DS4del*	30.23	9.49	55.77	0.00441
ALT (Log IU/L)	Patient’s age	1.46^a^	0.88	2.05	0.00001
	Gender (female)	−28.49	−39.27	−15.71	0.00008
	Cirrhosis	26.16	−4.94	66.63	0.10860
	*KIR2DS4del*	34.23	7.3	67.15	0.01004

**Fig. 4:**
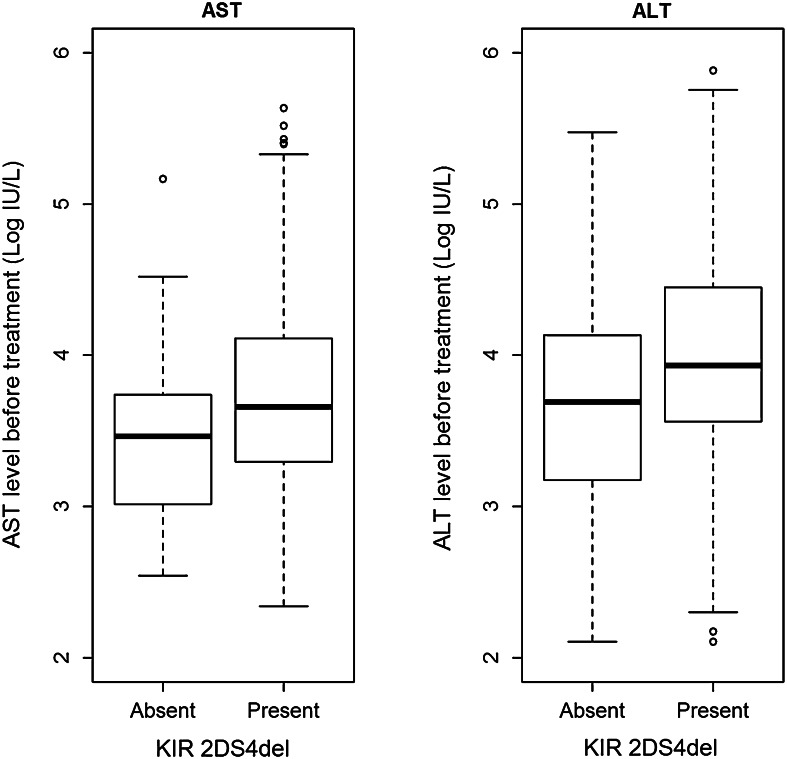
AST and ALT levels before therapy in *KIR2DS4del*-positive and *KIR2DS4del*-negative patients. Medians: first and third quartiles presented

Our analysis revealed an association of a *KIR* gene, the *KIR2DS4* variant with a 22 base pair deletion (*KIR2DS4del*), with activities of liver enzymes AST and ALT in patients before therapy (Table 4; Fig. 4). Both enzymes had values about 30 % higher in *KIR2DS4del*^+^ than in *KIR2DS4del*^−^ individuals (30.2 % for AST, *p* = 0.00441 and 34.2 % for ALT, *p* = 0.01004). These relationships were adjusted for other important characteristics, i.e. age of the patient, viral load, cirrhosis, and presence of *KIR2DS3* and *KIR2DS5* genes, found by us to influence viremia level (Kuśnierczyk et al. 2015). We did not find any other statistically significant association between clinical parameters and KIR genes.

**Fig. 5:**
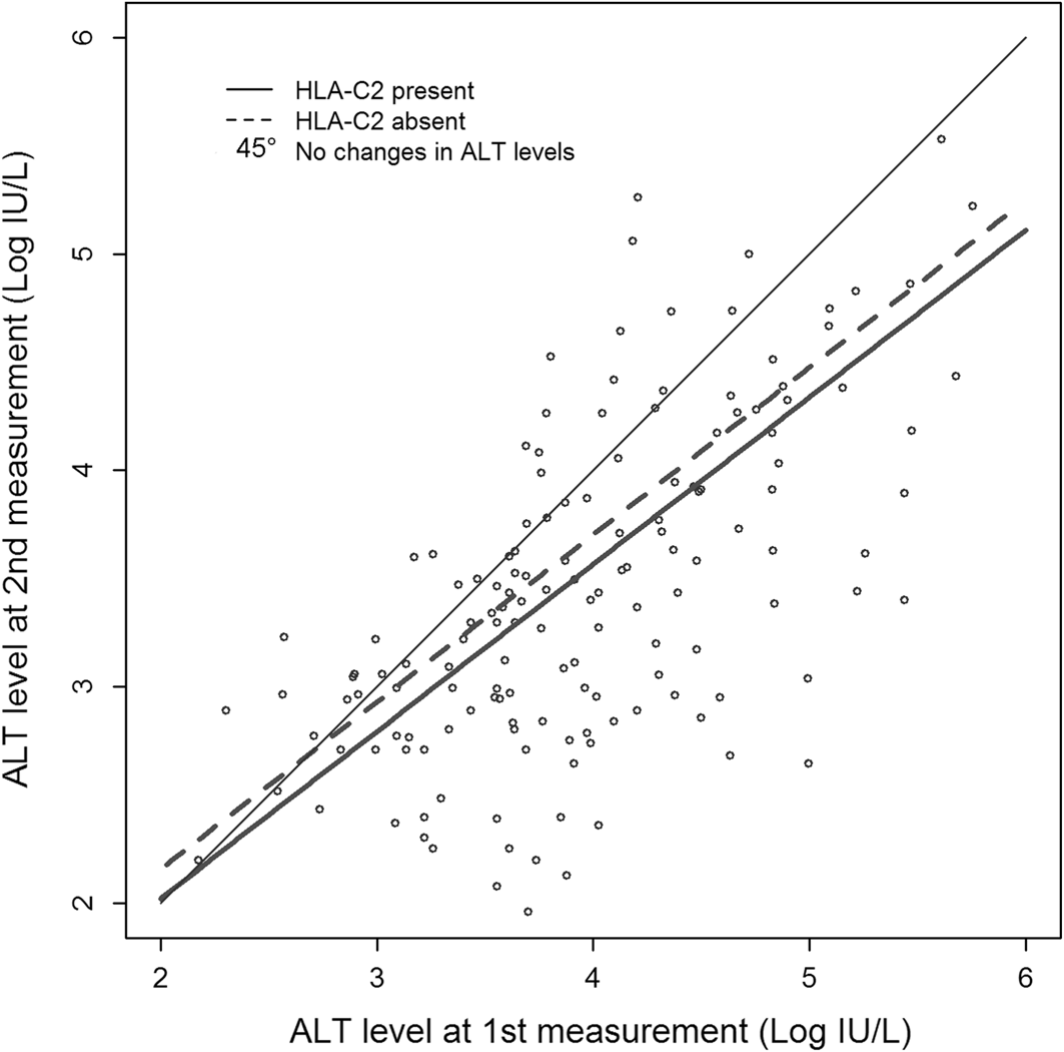
ALT levels before and after therapy of chronically HCV-infected patients depending on the presence or absence of HLA-C C2 allotype. A 45° *line* shows ALT levels expected if they did not decrease after therapy

Finally, ALT level month after therapy completion was clearly dependent on its value before therapy: patients with higher ALT values before therapy had also higher values after it. For example, patient with 1 % higher ALT before therapy had about 0.8 % higher level of ALT after treatment. Also, patient with 50 % higher level of ALT before had about 37 % higher level of ALT after therapy compared to patient with 50 % lower level of ALT before therapy (*p* < 0.00001). ALT values after therapy were also associated with *HLA*-*C C2* marker: decrease of ALT levels after therapy were stronger in case of *C2*^+^ individuals (−12 % on average) compared to *C2*^−^ patients (Fig. 5). In other words, in case of two groups of patients *C2*^+^ and *C2*^−^, with the same average level of ALT before therapy, the average level of ALT after treatment was lower than before therapy in both groups, but in case of *C*2^+^ patients it was additionally about 12 % lower comparing to *C2*^−^ ones. This result was adjusted for other clinical factors, age and gender, and all of them were nonsignificant.

## Discussion

Results of this study indicate that the outcome of HCV-infected liver disease and response to antiviral therapy are associated with various personal, genetic and biochemical data. The cohort of patients studied here was typical for the Polish population, including predominance of HCV 1b genotype of infectious virus (Panasiuk et al. 2013). EVR is a valuable parameter of the reaction of patient to treatment (Bura et al. 2012; Rao et al. 2014). We noticed that appearance of EVR was strictly dependent on the time lag between infection and start of treatment, what was also observed by others (Gupta and Singh 2012) and references therein). It is also known, that long-lasting chronic HCV infection is resistant to anti-viral treatment (Jang and Chung 2011) the observation confirmed also in our study.

Moreover, we have shown by statistical approach that each subsequent year of untreated HCV infection decreased the chance of EVR formation for 20 % (OR = 0.802). This is a clear indication that delayed access to antiviral therapy is unfavorable to patient. This raises the problem of early diagnosis, especially when we know that acute HCV infection is usually mild and asymptomatic.

Another interesting aspect of the current study was patient’s gender. Women were found to have twice higher chance than men to obtain EVR, in spite of having the same duration time before treatment (OR = 2.395), what may be seen from the Table 3, Figs. 2 and 3. It has also been shown that late liver damage in women was found to be less pronounced than in men, what was explained by long-term exposure to estrogens (Di Martino et al. 2004).

We also looked for the links between activity of liver enzymes and the progression of HCV liver disease. There was statistically significant association between AST activity and advancement of liver cirrhosis (Table 4). On the contrary, ALT activity did not show such links. It is known, however, that AST behavior is the reflection of liver damage (as it is manifested in heavy drinkers), while ALT is mainly a marker of functional disturbances of liver but, to a lesser degree, of its injury (Carrión et al. 2007; Iwata et al. 2013).

On the other hand, in the current study, high ALT levels were associated with EVR. It was shown by others (Dogan et al. 2013; Kim et al. 2012) that rapid fall of high ALT values correlates with the likelihood of achieving SVR.

Our result concerning *KIR2DS4del* gene, which was associated here with the levels of liver enzymes ALT and AST, also seems intriguing. This allele encodes an incomplete molecule, unable to anchor in the cell membrane, and therefore only a soluble molecule may be produced. This molecule has been proven to be nonfunctional, as it did not bind ligands of the full-length KIR2DS4 protein (Graef et al. 2009), although we cannot exclude the possibility that it binds additional ligands. A possible simple explanation, therefore, might lay in the lower frequency of full length *KIR2DS4* allele when the *KIR2DS4del* allele frequency is increasing (Gonzalez-Galarza et al. 2011; Kuśnierczyk et al. 2015). Nevertheless, in hepatitis B virus infection in the Chinese, both full-length and deleted alleles of *KIR2DS4* were associated with hepatocellular carcinoma; the presence of both of them was required for maximal effect (Pan et al. 2011). Similarly, the presence of both types of *KIR2DS4* alleles gave highest risk of kidney graft rejection, particularly in recipients with glomerulonephritis as a cause of kidney failure (Nowak et al. 2012). These findings suggest a biological role for the truncated KIR2DS4 protein, supported by a *KIR2DS4del* to *KIR2DS4*-*full*-*length* frequency ratio of approximately 2:1 in Caucasian populations (Gonzalez-Galarza et al. 2011), implying a positive selection for a defective allele. Therefore, soluble KIR2DS4 may play some role in the functioning of the immune system, although it has been shown not to compete for ligand binding with full-length KIR2DS4 (Graef et al. 2009), as mentioned above. Alternatively, the *KIR2DS4del* variants may be in strong linkage disequilibrium with a gene truly associated with liver functional disturbance and damage in our patients (manifested by raised ALT and AST levels, respectively), with HBV-induced hepatocellular carcinoma, and with renal transplant rejection. This, however, seems unlikely, because different *KIR2DS4del*-positive haplotypes may contain different alleles (KIR2DS4*003, 004, 006, 007), being in linkage disequilibrium with different alleles of this putative causative gene, particularly in genetically distant populations such as Chinese and Poles. *KIR2DS4* gene, without determining full-length versus deleted variants, was found protective against HCV infection, elevated ALT levels and cirrhosis in Argentinian Caucasians (Paladino et al. 2007) and in exposed intravenous drug users of Puerto Rican descent (Zúñiga et al. 2009). We have also described recently a correlation between ALT and AST levels before therapy and expression of another activating NK cell receptor, NKG2D, in livers of HCV-infected individuals (Mozer-Lisewska et al. 2014). In that study, we found a higher abundance of NKG2D^+^ cells than that of CD56^+^ cells in liver infiltrates, which suggests that other cells, presumably T cells, expressed NKG2D in addition to NK cells. T cells may also express activating KIRs, among them KIR2DS4 (Yen et al. 2001). KIR^+^ T cells expand during cytomegalovirus reactivation (Chan et al. 2013). It is, therefore, possible that such cells expand also in chronic HCV infection, contributing to *KIR* association with AST and ALT levels.

The ALT levels in our patients seemed to be influenced also by the presence of C2, but only after therapy. C2 is a ligand for KIR2DL1 and KIR2DS1; some C2 allotypes also interact with KIR2DL2 and KIR2DL3, as well as with full-length KIR2DS4 protein (Graef et al. 2009; Parham et al. 2012). The frequency of the *KIR2DL2* and *KIR2DL2* plus *HLA*-*C* was found higher in chronic hepatitis C patients than in control group (de Vasconcelos et al. 2013). The *KIR2DL1* and *KIR2DL3* genes were present in the great majority of our patients and controls (Kuśnierczyk et al. 2015); therefore, their possible associations, together with C2, with the response to treatment were difficult to detect.

In summary:

1. Duration time before the antiviral treatment as well as patient’s gender have significant impact on the appearance of EVR to therapy
2. Mean ALT and AST activities before treatment were higher in patients positive for defective *KIR2DS4* gene (*KIR2DS4del*)
3. A decrease of ALT activity after treatment was higher in *HLA*-*CC2*-positive individuals.
